# Anodal transcranial direct current stimulation of right temporoparietal area inhibits self-recognition

**DOI:** 10.3758/s13415-016-0461-0

**Published:** 2016-09-21

**Authors:** Sophie Payne, Manos Tsakiris

**Affiliations:** 1 Laboratory of Action & Body, Department of Psychology, Royal Holloway, University of London, Egham, Surrey, TW20 0EX London UK; 2 Department of Psychology, Royal Holloway, University of London, Egham, Surrey, TW20 0EX London UK

**Keywords:** Self–other discrimination, Self-recognition, Face recognition, Social cognition, Temporoparietal junction, tDCS

## Abstract

Self–other discrimination is a crucial mechanism for social cognition. Neuroimaging and neurostimulation research has pointed to the involvement of the right temporoparietal region in a variety of self–other discrimination tasks. Although repetitive transcranial magnetic stimulation over the right temporoparietal area has been shown to disrupt self–other discrimination in face-recognition tasks, no research has investigated the effect of increasing the cortical excitability in this region on self–other face discrimination. Here we used transcranial direct current stimulation (tDCS) to investigate changes in self–other discrimination with a video-morphing task in which the participant’s face morphed into, or out of, a familiar other’s face. The task was performed before and after 20 min of tDCS targeting the right temporoparietal area (anodal, cathodal, or sham stimulation). Differences in task performance following stimulation were taken to indicate a change in self–other discrimination. Following anodal stimulation only, we observed a significant increase in the amount of self-face needed to distinguish between self and other. The findings are discussed in relation to the control of *self* and *other* representations and to domain-general theories of social cognition.

In recent years, the neural underpinnings of self–other discrimination have been extensively investigated as part of the larger social neuroscience program. In particular, the study of self–other distinction was extended from the field of self-awareness to that of social cognition as it became clear that the process whereby the self is represented as distinct from others is a prerequisite for fundamental social-cognitive processes, such as empathy (Bird & Viding, 2014; Decety & Lamm, 2007). Improved understanding of the mechanisms involved in self–other discrimination may provide valuable insight into higher-level metacognition.

The right hemisphere has been extensively implicated in self-related processing, and more specifically in the discrimination of self from others (Decety & Sommerville, 2003; Feinberg & Keenan, 2005). One aspect of self–other discrimination that has been particularly lateralized to the right hemisphere is that of self-face recognition (Devue & Brédart, 2011; Keenan, Nelson, O’Connor, & Pascual-Leone, 2001; Keenan, Wheeler, Gallup, & Pascual-Leone, 2000; Uddin, Kaplan, Molnar-Szakacs, Zaidel, & Iacoboni, 2005). The ability to recognize our own face has been claimed to be fundamental to self-awareness (Gallup, 1970; Rochat & Zahavi, 2011), upon which the ability to recognize the self as existing among similar others can develop (Zahavi & Roepstorff, 2011). This process is crucial for complex forms of self-identity and social interaction (Povinelli & Simon, 1998). Among the specific brain regions implicated in self-face recognition, the causal involvement of the temporoparietal junction (TPJ) area has been explored with low-frequency repetitive transcranial magnetic stimulation (rTMS), used to transiently impair typical neural functioning. Following the application of rTMS to right inferior parietal lobule (IPL; encompassed within the temporoparietal area), Uddin, Molnar-Szakacs, Zaidel, and Iacoboni (2006) reported a disruption in the ability to discriminate one’s own face from the face of another. Participants were more likely to identify morphed faces containing 60 % of the other’s face, and only 40 % of their own face, as resembling their own face following stimulation. In line with this finding, after applying rTMS to the right TPJ, Heinisch, Dinse, Tegenthoff, Juckel, and Brüne, (2011) and Heinisch, Krüger, and Brüne (2012) showed that participants discriminated between their own and a familiar face (in a morphing video) at a point at which less of their own features were visible than before the stimulation. This was apparent in both self-to-other and other-to-self directions of morphing video. Taken together, these findings indicate that disruption of normal brain activity in the right TPJ facilitates self-face recognition, as participants are able to recognize their own face at an earlier point in a self–other morph—that is, when less of their facial features are actually visible than preceding stimulation. These findings indicate an active involvement of this region in self–other discrimination.

Neuroimaging evidence has highlighted an overlap between the regions within the right TPJ involved in performing high-level social-cognitive tasks, including theory of mind (Frith & Frith, 2006), empathy (Jackson, Brunet, Meltzoff, & Decety, 2006), and perspective taking (Aichhorn, Perner, Kronbichler, Staffen, & Ladurner, 2006), with those involved in lower-level self–other processing (Decety & Lamm, 2007). This has led to the suggestion that a domain-general computational mechanism associated with low-level agency processing (comparing signals arising from the self with externally produced signals) may support higher-level social-cognitive processes (Decety & Lamm, 2007). Such a mechanism would allow an individual to distinguish between his or her own and another’s perspective, thus supporting processes such as empathy and theory of mind.

As previous research indicated, disruption of activity in the right TPJ area results in a bias toward self-recognition. In terms of social cognition, a heightened sense of self and a reduction in sensitivity toward the other may alter the self–other distinction and inhibit the ability to understand another’s mental state or perspective. If a low-level self–other discrimination mechanism does support higher-level processing, then enhancing this mechanism could result in improved social-cognitive processing by facilitating the recognition of external, other-related signals. On a lower-level self–other discrimination task, this might appear as a facilitation of other-recognition and an inhibition of self-recognition.

Santiesteban, Banissy, Catmur, and Bird (2012) have shown that higher-level social-cognitive processing can be enhanced through a brief period of anodal transcranial direct current stimulation (tDCS) over the wider right temporoparietal area. Following 20 min of anodal tDCS, participants performed significantly better at two social-cognitive tasks than did those participants receiving cathodal stimulation over the same area. Application of tDCS involves the modulation of cortical excitability by passing a direct current through the cortex (between an anodal and a cathodal electrode). Anodal stimulation causes depolarization of the resting membrane potential, making the neurons under the electrode site more likely to fire, whereas cathodal stimulation leads to hyperpolarization, resulting in decreased neuronal excitability (Nitsche & Paulus, 2000). This type of stimulation can lead to sustained modulations in cortical excitability, with the duration of the aftereffect being dependent on the intensity and duration of the stimulation period (Nitsche & Paulus, 2001). This technique can provide insight into the role of cortical regions in cognitive functions by modulating neural excitability, and the corresponding effects of the stimulation on the targeted processes can be observed. Santiesteban et al.’s (2012) findings could reflect facilitation of the mechanism suggested to underlie both higher-level and low-level social-cognitive processing. If so, the same type of stimulation over the same area should also affect the ability to discriminate between one’s own and another’s face. Furthermore, this may appear behaviorally as an inhibition of self-recognition, as the processing of external, other-related signals is facilitated. No study to date has investigated the role of the wider temporoparietal area in self–other face discrimination with tDCS. Such an investigation, coupled with previous tDCS research into the role of this area in higher-level social cognition, would provide insight into this area’s contribution to a potentially domain-general mechanism spanning both high- and low-level social-cognitive processes.

In our experiment, we aimed to investigate the role of the right temporoparietal area in low-level self–other discrimination by targeting this region with anodal tDCS. We adopted an established face-morphing task (Heinisch et al., 2011; Heinisch et al., 2012; Keenan, Freund, & Hamilton, 2000; Tsakiris, 2008) to investigate the extent to which anodal stimulation over the right temporoparietal area would affect the ability to discriminate self from other. Participants watched videos of their own face morphing into the face of a familiar other, and of a familiar face morphing into their own (both before and after 20 min of tDCS) and responded when they detected a change in the identity of the face in the video. The pre-tDCS block of videos acted as a baseline measure of self–other discrimination, and changes in performance from pre-tDCS to post-tDCS were compared. On the basis of the results of Uddin et al. (2006) and Heinisch et al. (2011; Heinisch et al., 2012), which showed that impaired functioning of right TPJ facilitated self-recognition by reducing the amount of self-face required to discriminate between self and other, and the results of Santiesteban et al. (2012), showing enhanced social-cognitive ability following anodal tDCS to the right temporoparietal area, we hypothesized that an increase in cortical excitability in the right temporoparietal area would inhibit self-recognition by requiring more of the self-face to be visible in order to discriminate between self and other.

## Material and method

### Participants

Sixty (44 female, 16 male; mean age 21.54 years, SD = 4.54) participants volunteered to take part in the study and were reimbursed for their time. All participants were screened for possible contraindications to tDCS and provided signed informed consent for their participation. Once recruited, participants were randomly assigned to the anodal (*n* = 20: 15 female, five male), cathodal (*n* = 20: 15 female, five male), or sham (*n* = 20: 14 female, six male) stimulation group (see Table 1). We chose this number of participants per condition on the basis of the reported *N* sizes in recent, similar tDCS studies (see Enticott et al., 2012; Santiesteban et al., 2012). All participants were naïve to the purpose of the study and were unaware of the type of stimulation they received until after the experiment. The study was approved by the Royal Holloway, University of London Ethics Committee.

**Table 1:** Demographic data following the exclusion of three participants

	Demographic Data					
	Age	Gender		Handedness		
	*M* (*SD)*	Female	Male	Right	Left	Ambidextrous
Anodal	19.6 (1.23)	15	5	18	2	–
Cathodal	22.25 (5.97)	14	6	20	–	–
Sham	22.45 (4.41)	12	5	14	2	1

### Design

The study utilized a double-blind, sham-controlled, mixed design, with the within-subjects factor Timing of the Face-Morphing Task (pre- vs. poststimulation) and the between-groups factor tDCS Group (anodal vs. cathodal vs. sham). We chose a between-groups design to avoid learning effects on the video-morphing task across several sessions of stimulation. Participants took part in one experimental session in which they completed two blocks of the video-morphing task, separated by 20 min of tDCS.

### Stimuli

We used a modified version of Keenan, Freund, and Hamilton’s (2000) video-morphing task (see also Heinisch et al., 2011; Heinisch et al., 2012; Tajadura-Jiménez, Grehl, & Tsakiris, 2012). First, a photograph was taken of each participant’s face with a neutral expression. Participants with glasses or facial hair did not take part, in order to control variation in the morphing videos. All photographs were converted to grayscale, flipped horizontally so as to reflect the orientation of the self-face that participants would be most accustomed to seeing (from mirror exposure), and a template was applied around the face to remove hair and nonfacial features. All photo manipulation was completed using Adobe Photoshop CS6. Every participant was then paired with a familiar, gender- and skin-tone-matched famous individual (following the procedure of Heinisch et al., 2011; Heinisch et al., 2012, participants indicated the name of a famous individual with whom they were highly familiar), and the same procedure was applied to a photograph of the famous face. Famous faces were used in light of the finding that rTMS over right TPJ only affects discrimination of the self from familiar, but not from unfamiliar, faces (Heinisch et al., 2012). The face-morphing software Abrasoft Fantamorph (www.fantamorph.com) was used to create a morphing continuum between the two faces, and 100 images representing 1 % steps of morphing between the two faces were exported. Adobe Premier Pro was used to convert the image series into two directions of video (self to other and other to self) with three durations (10, 15, and 20 s), resulting in six videos for each participant. The three durations and two directions of video were created to make the videos less predictable, thus requiring participants to make a conscious choice regarding when the face in the video started to look more like the individual it was morphing into, rather than responding at the same temporal point in each video.

### tDCS parameters

Participants were stimulated with either the anodal or cathodal electrode placed over right temporoparietal area (electrode position CP6: electroencephalographic [EEG] 10/20 system; Herwig, Satrapi, & Schönfeldt-Lecuona, 2003) and the reference electrode over the Vertex (measured individually for each participant). Stimulation was delivered via two 3.5 cm^2^ electrodes, placed within saline-soaked sponges, for 20 min at the intensity of 1 mA (30-s ramp-up, 20-s ramp-down). The setup for the sham stimulation was identical, with the anodal electrode position being counterbalanced across participants, except that the stimulator was only switched on for the first 15 s of stimulation. Participants were asked following the experiment if they were aware of the type of stimulation they received, with the majority being unaware. The electrode montage and tDCS parameters were identical to those used previously to successfully modulate the cortical excitability of right TPJ (Santiesteban et al., 2012).

### Video-morphing task

**Fig. 1:**
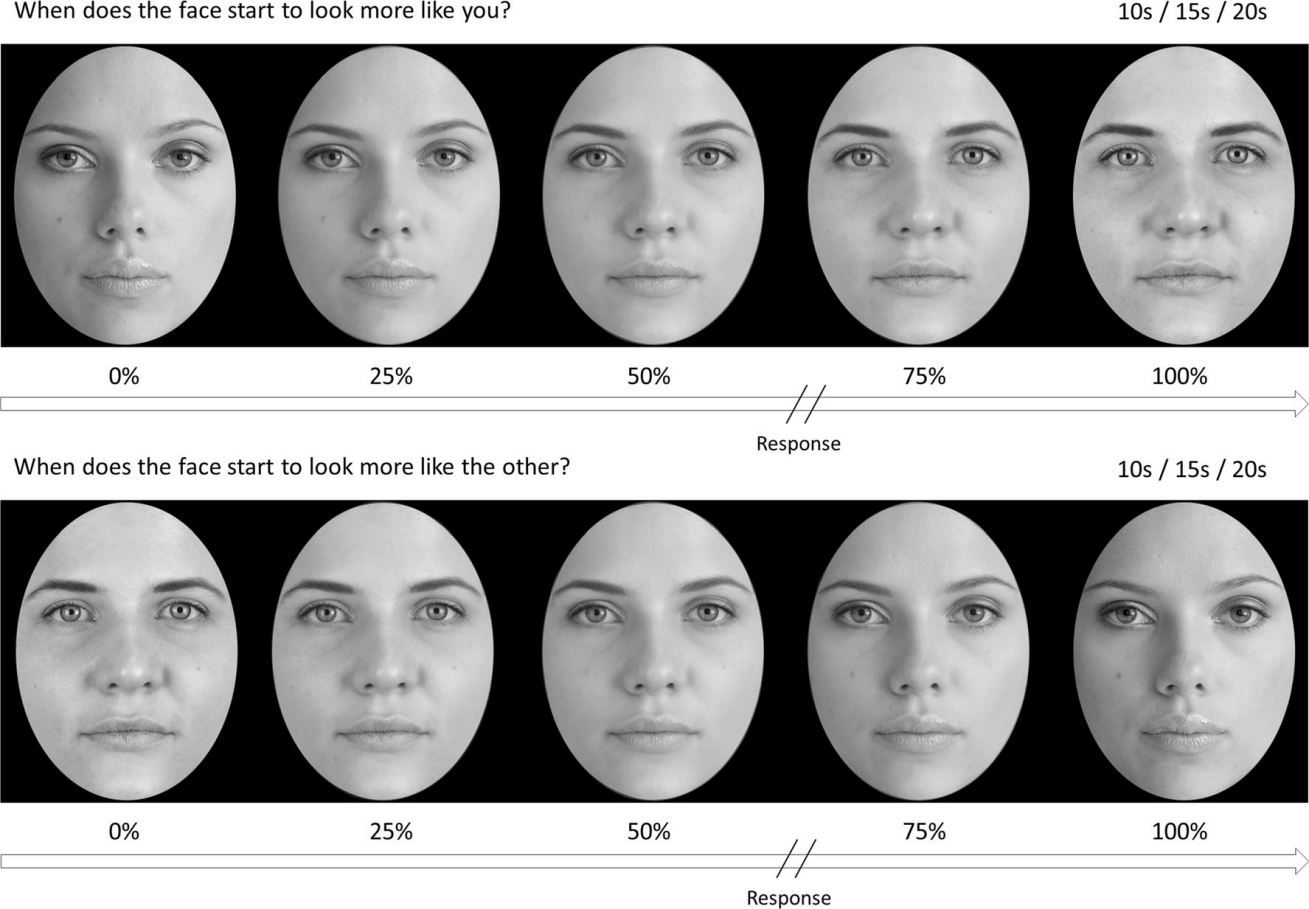
Examples of the two directions of morphing video used in the experiment. The videos were 10, 15, or 20 s in length and morphed either from a familiar face into the participant’s own face (top), or from the participant’s face into a familiar face (bottom).

The video-morphing task consisted of videos of the participant’s face morphing into a famous face, or the same famous face morphing into the participant’s own face. Each of the six videos was presented five times per block in a randomized order, resulting in 30 trials per block. Before each video, a fixation cross was presented on screen for 1–2.5 s. The video was then presented, and participants watched the video and made their response. Participants were told to respond with a buttonpress as soon as they detected a change in the identity of the face in the video, such that, when the video morphed from self to other, participants responded when the face started to look more like the familiar other, and when the video morphed from other to self, participants responded when the face started to look more like their own (see Fig. 1). All participants responded with their right index finger. The prestimulation video-morphing trials provided a baseline measure of the “point of discrimination” between self and other, and we compared performance in the post-tDCS block with the baseline performance to investigate the effect of tDCS on self–other discrimination.

### Data analysis

Preceding the analysis, the raw response time data were converted into percentages of self-face present in the morphing video at the point at which participants judged a change in identity. Because past research employing brain stimulation to investigate self–other discrimination (i.e., TMS to disrupt cortical functioning) had affected this ability independently of the direction of the morphing video (self to other vs. other to self; Heinisch et al., 2011; Heinisch et al., 2012), and because we were not interested in the directions themselves, but rather in the amount of the self-face required to discriminate between the two faces, the data were averaged across durations and directions of the video to create the points of discrimination before and after tDCS, reflecting the amount of self-face needed to discriminate between the self and another’s face.^1^ Following this, the data were cleaned by identifying participants with responses outside two standard deviations from the mean response. Three participants were excluded from the analysis, leaving a total of 57 data points for analysis. See Table 1 for the demographic data.

## Results

The pre-tDCS and post-tDCS self–other discrimination performance was entered into a repeated measures analysis of variance with tDCS Group as a between-subjects factor and Timing of Task (pre- vs. post-tDCS) as a within-subjects factor. We found no significant effect of either the timing of the task, *F*(1, 54) = 2.34, *p* = .13, *η* ^2^ = .04, or tDCS group, *F*(2, 54) = 0.69, *p* = 51, *η* ^2^ = .03. However, the interaction between timing of task and tDCS group was significant, *F*(2, 54) = 5.18, *p* = .009, *η* ^2^ = .16.

**Fig. 2:**
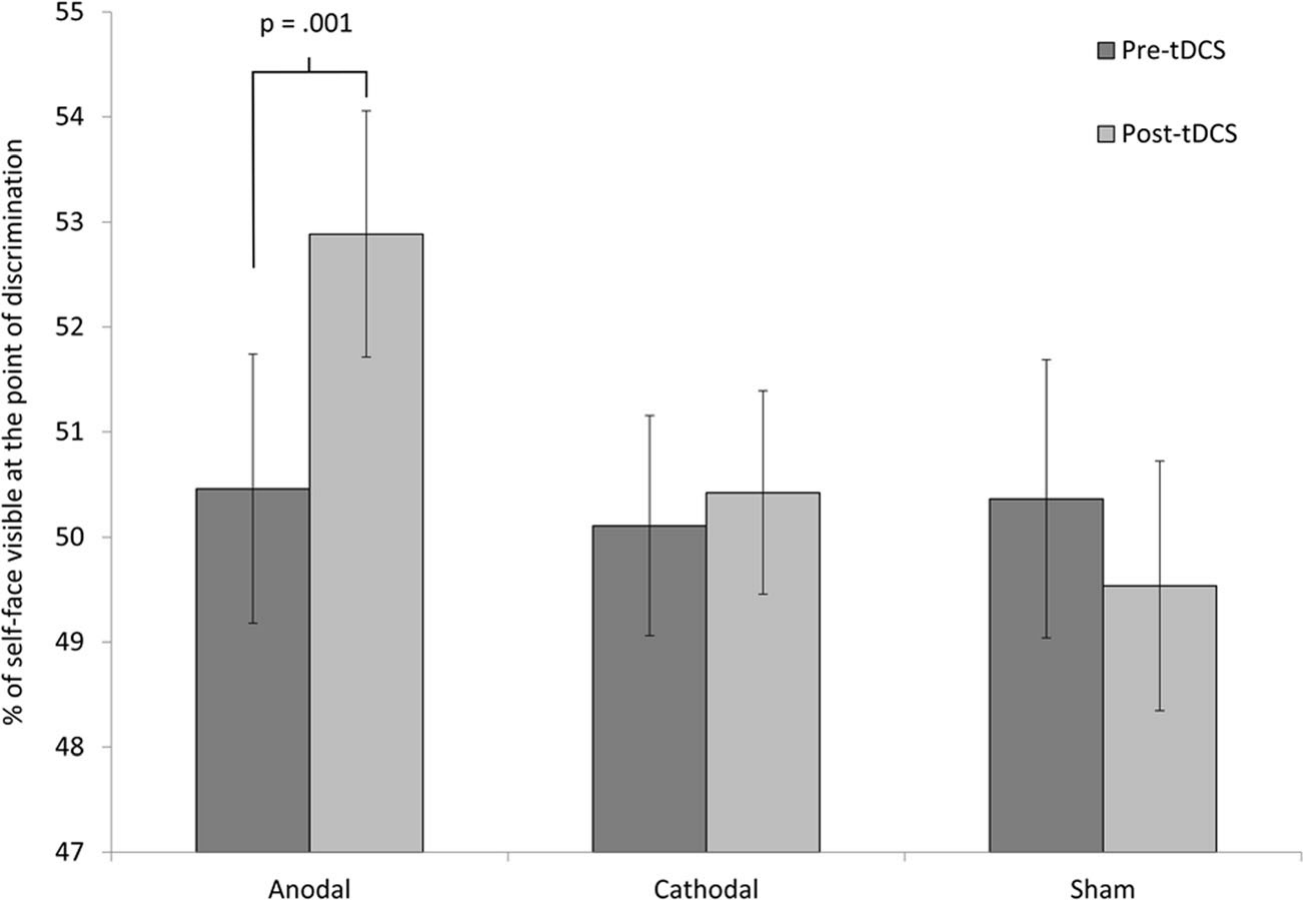
Mean percentages of self-face visible at the points of discrimination before and after transcranial direct current stimulation (tDCS). Error bars represent standard errors.

Post-hoc *t* tests, with Bonferroni correction applied for multiple comparisons, highlighted a significant increase in the percentage of self-face present at the point of discrimination following anodal tDCS: pre-tDCS *M* = 50.46, post-tDCS *M* = 52.88, *p* = .001. We also observed a slight increase following cathodal stimulation, and a slight decrease following sham stimulation; however, neither or these differences approached significance: cathodal pre-tDCS *M* = 50.11, post-tDCS *M* = 50.42, *p* = .68; sham pre-tDCS *M* = 50.36, post-tDCS *M* = 49.54, *p* = .30. See Fig. 2.

## Discussion

Previous research had investigated the effect of low-frequency rTMS over right TPJ on self–other discrimination. In our study, we sought to expand the understanding of the role of the right temporoparietal area in this process by observing the effects of excitatory anodal tDCS over this region on a self–other face discrimination task. Participants watched videos that morphed between their own and a familiar other’s face, and responded when they judged a change in identity of the face. The amount of the participant’s own face in the video at the point of response was used to reflect the “point of discrimination” between the participant and the familiar other. Participants performed this task before (baseline) and after a 20-min session of tDCS in which they received anodal, cathodal, or sham stimulation over the right temporoparietal area. Following anodal stimulation, but not cathodal or sham, we observed a change in the participants’ ability to discriminate between self and other. Specifically, following stimulation participants required more of their own face to be visible in order to discriminate between self and other. In effect, self-recognition was inhibited, since participants recognized fewer frames of the morphing video as resembling themselves.

Three previous studies have applied rTMS over right TPJ to investigate this area’s functional role in self–other discrimination. Following rTMS, participants were more likely to judge images containing 60 % of the other and only 40 % of the self as “self” (Uddin et al., 2006), and judged a change in identity in videos morphing between the self and the other at a point that contained more of the other’s face than before stimulation (Heinisch et al., 2011; Heinisch et al., 2012). In essence, the rTMS facilitated self-recognition, since more frames of the morphing video were recognized as self (rather than other) following rTMS to right TPJ (Heinisch et al., 2011; Heinisch et al., 2012; Uddin et al., 2006). The findings that self-recognition is facilitated when activity in right TPJ is disrupted with rTMS, and inhibited when neural excitability is enhanced with tDCS, as our results suggest, may seem surprising when considering the right hemisphere’s involvement in self-recognition. However, these results are in line with the idea that the right temporoparietal area is involved in a mechanism that distinguishes between representations of the self and other, underpinning higher-level social cognition (Santiesteban et al., 2012), as we explain below.

In embodied accounts of social cognition, control over online representations of the self and other are thought to form the fundamental basis for social-cognitive processes such as mentalizing and empathy. In a review of the literature, two recent accounts put forward a persuasive picture of functional–anatomical overlaps between higher-level social-cognitive processes (mentalizing) and lower-level self–other discrimination tasks (control of imitation and agency processing), speculating that the low-level computational mechanisms associated with self–other discrimination processes may play a domain-general role in self–other processing (Brass, Ruby, & Spengler, 2009; Decety & Lamm, 2007). For example, the outcome of a low-level agency judgment—that is, whether or not an observed action is attributed to the self or to another—could be applied in a more abstract sense to attribute a mental state to either the self or another (see Brass et al., 2009). In a similar vein, this mechanism could also extend to the sharing and understanding of another’s emotional states. In Bird and Viding’s (2014) self-to-other model of empathy, a crucial step in empathizing is the “self–other switch”: an active process in which the empathizer switches from focusing on the signals arising from the self to focusing on the state of the target individual. This idea is consistent with evolutionary views that higher-order processes operate on the framework of preceding levels of processing (see also Decety & Lamm, 2007). Bird and Viding speculated that the location of such a mechanism may be TPJ, due to its involvement in the control of *self* and *other* representations. If higher-order social cognition relies, at least in part, on the same basic computational mechanisms as low-level discriminatory processes, the active role of right TPJ in this process, suggested in our study, may be to inhibit representation of the self, in order to enhance representation and recognition of another.

This is in line with the idea that the default state of the cognitive representational system appears to be “self,” and that switching to a state in which the representation of another is enhanced is an active process (Bird & Viding, 2014; Gusnard, Akbudak, Shulman, & Raichle, 2001). Research suggests that the state of the self often influences judgments about others—an “egocentricity bias”—whether these be judgments about another’s beliefs (Nickerson, 1999), affective state (Silani, Lamm, Ruff, & Singer, 2013), or visual perspective (Surtees & Apperly, 2012). To accurately represent the state of another, online representations of the self must be inhibited. Santiesteban et al. (2012) demonstrated that anodal tDCS over the right temporoparietal area reduced the extent to which the participant’s own perspective interfered with taking the incongruent visual perspective of another person. In essence, the participant’s own perspective was inhibited, whereas the perspective of the other was enhanced. This finding may reflect the facilitation of such a representational-switching mechanism. Our results may reflect the operation of this same mechanism at a lower level, resulting in enhanced recognition of a familiar other’s face while inhibiting recognition of the self, lending weight to the idea that low-level computational mechanisms may support higher-level metacognition.

Although we might have expected to observe in the cathodal group an opposite effect from the one observed in the anodal group, the lack of an effect from cathodal stimulation is not entirely surprising. In a recent meta-analysis of tDCS studies, Jacobson, Koslowsky, and Lavidor (2012) reported that, whereas in the motor domain the majority of anodal stimulation leads to a facilitation, and cathodal stimulation leads to an inhibition, cathodal stimulation in the cognitive domain is unlikely to result in a decrease of function in cognitive processing. This has been attributed to compensatory processes in the complex neural networks involved in cognitive processes. In line with this theory, we did not find any differences in self–other discrimination following cathodal stimulation of the temporoparietal area, but instead observed a selective effect of anodal stimulation.

The stimulation site in our study was localized using the EEG 10/20 system. Although the preferable approach would have been to use fMRI-guided neural navigation to individually target right temporoparietal area for each participant, localization with the EEG 10/20 system is considered acceptable with this type of stimulation (Herwig et al., 2003). Future studies should also consider including an active control stimulation site, such as left temporoparietal area, to ensure that any effect of stimulation is site-specific. However, although this approach is considered acceptable to localize the stimulation site, it is important to note that it is unlikely that only the neural activity in right temporoparietal area was modulated by the tDCS in our experiment. Datta et al. (2009) have shown that tDCS delivered by rectangular sponge electrodes (such as those used in our study) results in diffuse modulation of neural activity that is not specific to the site targeted. Future research employing a ring electrode configuration, which has been shown to provide superior spatial focality, would provide valuable insight into the specificity of our effect to the modulation of neural activity in right temporoparietal area.

There is also a possibility that the effect we observed was influenced by uncontrolled variables such as baseline neural excitability. We chose to use a between-group design to avoid practice effects on the video-morphing task across several sessions; however, this introduced the possibility that the effect we saw poststimulation could have been due to differences in baseline neurological features between our three stimulation groups. Although we attempted to control for differences between the groups on baseline self–other discrimination ability through the pre-tDCS video-morphing task, the effect of tDCS on modulating neuronal excitability may have varied between the groups, despite the random allocation of participants to the tDCS groups in our study. However, it is unlikely that the baseline neural excitability of the individuals in the anodal group was consistently different from that of those in the other two groups. Future studies would benefit from providing baseline measures of neural excitability. It should also be noted that, although our study was comparable to previous tDCS research comparing the effects of stimulation across groups (Enticott et al., 2012; Santiesteban et al., 2012), our sample size and those of previous research were small due to between-subjects designs, which could mean that our results and those of previous research are statistically underpowered. Therefore, it is important that the present findings be interpreted with caution, and future research would benefit from attempting to replicate the previous findings with a larger sample size.

To conclude, our results could suggest that anodal tDCS-induced modulation of cortical excitability, targeting the right temporoparietal area, affects self–other face discrimination by inhibiting recognition of the self while facilitating the recognition of a familiar other. The present study provides some support for previous research indicating the functional involvement of the right temporoparietal area in self–other discrimination, and it provides insight into the active role of this region in this process. It has been speculated that the right temporoparietal area may support higher-order social-cognitive processes including mentalizing and empathy, and our study suggests that this region may support these processes by enhancing the representation of others and inhibiting the representation of the self.

## Footnotes

1 When the directions of morphing were analyzed separately, a main effect of direction of morphing was present, which might be expected, since inevitably there were different levels of familiarity between the two face identities: *F*(1, 54) = 309.49, *p* < .001, *η* ^2^ = .851. However, importantly, this factor did not significantly interact with tDCS Group in the either the two- or three-way interactions: Direction of Morphing × tDCS Group, *F*(2, 54) = 2.07, *p* = .14, *η* ^2^ = .071; Direction of Morphing × tDCS Group × Timing, *F*(2, 54) = 0.58, *p* = .57, *η* ^2^ = .021. We did find a significant interaction between timing and tDCS group (as in our collapsed analysis), *F*(2, 54) = 5.18, *p* = .009, *η* ^2^ = .161, suggesting that the effect of anodal tDCS was independent of the direction of morphing.
